# Minnesota

**Published:** 1899-11

**Authors:** L. Hay

**Affiliations:** Secretary


Secretary.

### MINNESOTA STATE VETERINARY MEDICAL ASSOCIATION.

The fifth semi-annual meeting of the Minnesota State Veterinary Medical Association was held at Faribault on July 13 and 14, 1899. The first session was called to order at 2 P.M. at the Arlington House, and the following members responded to roll-call: Drs. W. Amos, Owatonna; J. J. Annand, S. D. Brimhall, C. C. Lyford, M. J. Sexton, J. S. Butler, Minneapolis; R. Price, St. Paul; M. H. Reynolds, St. Anthony Park; L. Hay, Faribault; K. J. McKenzie, Northfield; S. H. Ward, St. Cloud; J. A. Hanisch, Zumbrota; A. F. Lee, Red Wing; J. N. Gould, Worthington; J. W. Gould, Fairmont.

Reports of secretary and treasurer were read and adopted.

President Reynolds then addressed the meeting, speaking at length on the subject of the many recent advances made by the profession.

The chairmen of the different committees were then called upon to make their reports, and Dr. Reynolds, as chairman of the Committee on Infectious Diseases, gave a very interesting report on dealing with tuberculosis in this State, stating that the demand for tuberculin has immensely increased within the last year.

When the committees had made their reports and other business of a routine nature had been disposed of the meeting adjourned to L. F. Miller's livery barn, where the following operations were performed by the different members: Trephining for third lower molar tooth, Dr. C. C. Lyford; trephining for third upper molar, Dr. J. N. Gould; trephining for third upper molar, Dr. J. G. Annand.

The meeting then adjourned until after supper. On reassembling the routine business was again taken up. Dr. Lee, of Red Wing, was elected to membership. A number of interesting cases were reported by different members.

Friday morning was spent in examining some obscure cases of lameness, after which the following operations were performed: Castration of a cryptorchid, Dr. Lee; operation for synovitis in the hind fetlock, Dr. C. C. Lyford; castration of another cryptorchid, Dr. K. J. McKenzie; plantar neurectomy, Dr. C. C. Lyford; same operation, Dr. S. D. Brimhall.

After dinner a short time was devoted to a discussion of Association business. A vote of thanks was tendered Dr. Hay for clinical material secured; to Mr. L. F. Miller, for his courtesy in supplying suitable quarters for holding the clinics, and to the proprietor of the Arlington Hotel for hospitality and courtesy to the members of the Association.

The meeting then adjourned.

L. Hay,
Secretary.

